# The Potential Role of Polyphenol Supplementation in Preventing and Managing Depression: A Review of Current Research

**DOI:** 10.3390/life14101342

**Published:** 2024-10-21

**Authors:** Mohd Farhan, Mohd Faisal

**Affiliations:** 1 Department of Chemistry, College of Science, King Faisal University, Al Ahsa 31982, Saudi Arabia; 2 Department of Basic Sciences, Preparatory Year, King Faisal University, Al Ahsa 31982, Saudi Arabia; 3 St. Michael’s Unit, Department of Psychiatry, Mercy University Hospital, Grenville Place, T12WE28 Cork, Ireland; 4 Tosnú Mental Health Centre, West Village, Ballincollig, P31N400 Cork, Ireland

**Keywords:** depression, polyphenol, clinical trials, supplement, antidepressant

## Abstract

Depression is a common mental illness that affects 5% of the adult population globally. The most common symptoms of depression are low mood, lack of pleasure from different activities, poor concentration, and reduced energy levels for an extended period, and it affects the emotions, behaviors, and overall well-being of an individual. The complex pathophysiology of depression presents challenges for current therapeutic options involving a biopsychosocial treatment plan. These treatments may have a delayed onset, low remission and response rates, and undesirable side effects. Researchers in nutrition and food science are increasingly addressing depression, which is a significant public health concern due to the association of depression with the increased incidence of cardiovascular diseases and premature mortality. Polyphenols present in our diet may significantly impact the prevention and treatment of depression. The primary mechanisms include reducing inflammation and oxidative stress, regulating monoamine neurotransmitter levels, and modulating the microbiota–gut–brain axis and hyperactivity of the hypothalamic–pituitary–adrenal (HPA) axis. This review summarizes recent advances in understanding the effects of dietary polyphenols on depression and explores the underlying mechanisms of these effects for the benefit of human health. It also highlights studies that are looking at clinical trials to help future researchers incorporate these substances into functional diets, nutritional supplements, or adjunctive therapy to prevent and treat depression.

## 1. Introduction

It is widely acknowledged that major depressive disorders are among the leading causes of disability on a global scale. Nearly 5% of the world’s illness burden is attributable to depression, which the World Health Organization (WHO) estimates to affect 322 million people worldwide [1,2]. Depressive disorder or a depressive episode and dysthymia, a chronic type of mild–moderate depression, are the two primary subtypes of depression. The onset of symptoms in both circumstances significantly impairs psychosocial functioning, lowers life quality, and heightens the risk of suicide actions [3,4]. The precise cause of depression and related diseases is still unknown, despite extensive and persistent investigation. Environmental, genetic, and physiological variables, including early and adult life stressors (biological, psychological, and social), are believed to contribute to the complex molecular pattern underpinning depression symptoms [5].

All around the globe, people’s mental health has taken a major hit due to the COVID-19 epidemic [6]. During this pandemic, many have experienced significant stress due to the loss of loved ones, fear of infection, isolation, unemployment, homeschooling, and financial worries. These factors have heightened feelings of anger and loneliness and contributed to the increase in the prevalence of mental disorders like depression. The WHO reports that the prevalence of symptoms associated with depression has surged by 25% in recent times [6,7]. Depression is the most economically burdensome mental illness [8] and is a major comorbidity in many debilitating conditions [9], including cardiovascular disease, diabetes, rheumatoid arthritis, cancer, and many more. Consequently, this mental illness is a major focus in healthcare systems all over the globe [6]. In light of this, it is glaringly obvious that we must discover means of promptly attending to people’s mental health; the need for innovative antidepressant drugs has increased over the past several years [6,10].

Compared with other organs, the brain seems to be more vulnerable to free radical assaults. The function of serotonergic and catecholaminergic receptors can be impacted by an excess of free radical species, which can damage the phospholipids in neuronal membranes [11]. A change in the redox state has been observed in the central nervous system of patients with depression, and oxidative abnormalities have been linked to decreased levels of certain endogenous antioxidative enzymes [12]. Although there is evidence that pharmacological treatments can alleviate moderate to severe depression symptoms, the effect sizes of these treatments are small. Around 30% of individuals with depression do not show sufficient improvement with these current drug regimens. In addition, some adverse effects are linked to antidepressants [13].

Mainstay treatments for mental health issues like depression include psychotherapy, medication, and social interventions, but these approaches have drawbacks, such as low tolerance and unwanted side effects from psychotropic medications (i.e., sexual dysfunction, insomnia, anxiety, and dry mouth) [6], and it may take time to establish a therapeutic alliance during psychotherapeutic interventions. For this reason, finding new ways to treat and prevent these diseases is crucial. Sunlight and physical activity may alleviate depression symptoms and reduce the likelihood of developing the disorder, according to multiple research studies [14,15,16]. In addition, the number of studies investigating the potential antidepressive effects of minerals and natural items in the diet is currently increasing rapidly. More and more research is pointing to the potential anti-inflammatory and anti-oxidative properties of natural foods like medicinal plants, fruits, and vegetables, as well as their ability to enhance neurological function and modulate biomarkers and signaling pathways linked to depression [6,17,18].

**Figure 1:**
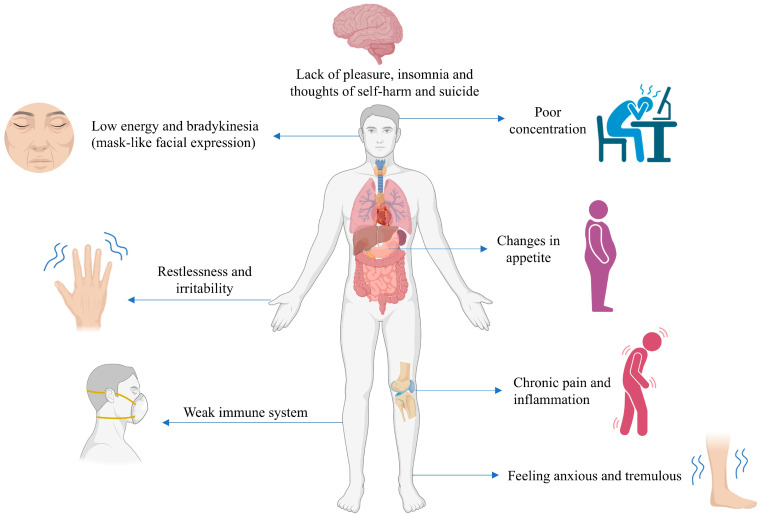
Typical signs and symptoms of depression.

Many studies have looked into the pharmacological effects of flavonoids, which are polyphenols found naturally [19]. There are many ways in which flavonoids, which are included in nearly all foods, including fruits, cereals, vegetables, alcohol, and tea, might prevent or reverse stress [20]. Polyphenols, particularly flavonoids, have been shown to have a broad range of therapeutic effects, such as anti-inflammatory, antioxidative, immunomodulatory, anti-carcinogenic, cardioprotective, and neuroprotective activity [20]. The antidepressive effects of natural chemical substances, particularly flavonoids, which have several effects on the brain, have received a substantial amount of attention throughout the past few decades [20,21]. It has been shown recently that polyphenol supplementation effectively improves depressive symptoms. Physical illness can serve as a contributing factor that exacerbates depression, indicating the necessity for preventive supplementation to improve depressive symptoms. Different types of polyphenols may exert distinct effects, indicating that various populations experiencing depression could derive benefits from specific polyphenols [20]. Animal models of depression in many preclinical investigations have demonstrated that certain flavonoids can alleviate symptoms of depression. Some hypothesized mechanisms for their antidepressive activity include an increase in neurotrophic factors, neurogenesis, and expression levels of different neurotransmitters in the brain [19,22]. This review outlines the existing knowledge and mechanisms of action based on preclinical and clinical studies, focusing on the antidepressive effectiveness of specific polyphenols. The objective of this article is to compile a list of major polyphenols exhibiting potential antidepressive properties from the existing literature, with the aim of facilitating the development of more effective nutraceutical products with minimal side effects for the alleviation of human depression. The major symptoms of depression are highlighted in Figure 1.

### Methodology

**Figure 2:**
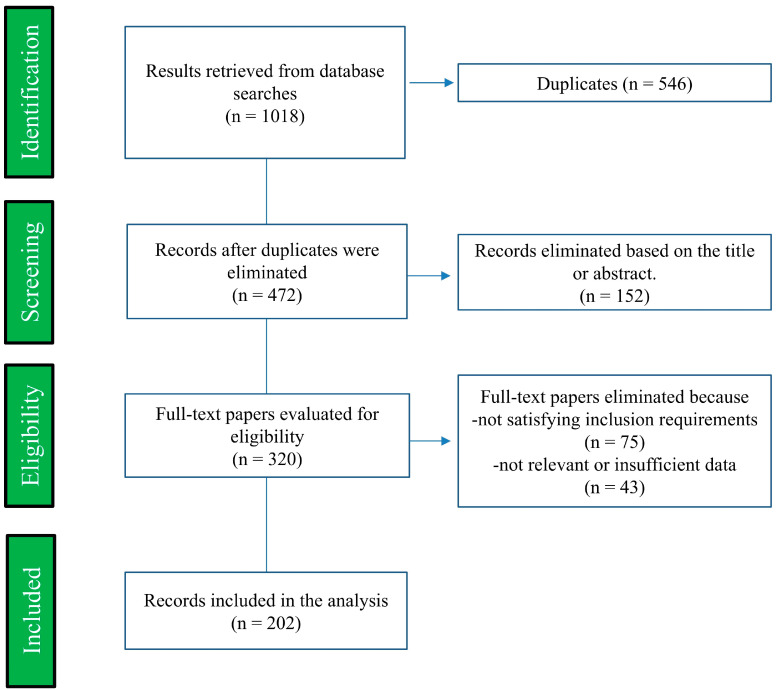
Flowchart illustrating the approach for the review.

The following databases were searched for relevant literature: Scopus, PubMed, and Web of Science. This meta-analysis incorporated research published up until 30 May 2024 that evaluated the influence of polyphenols on depressive symptoms in either observational or clinical investigations. Incorporating publications that calculated dietary polyphenol content or supplement dosages into our database was a priority. The study selection process is shown in Figure 2. The database search yielded a total of 1018 records. After eliminating 546 duplicate articles, we screened 472 studies and excluded 152 based on their titles and/or abstracts. We read the full texts of the 320 eligible studies, excluded 75 for not meeting the inclusion criteria, and removed 43 for being irrelevant or having insufficient data. A total of 202 studies concentrating solely on the effects of polyphenols on health and depression and having relevant data were examined. Review articles and original research based on animal studies and clinical trials were given top priority. Research lacking a depression rating scale, as well as those published in non-English languages or utilizing secondary data sources (such as reviews, meta-analyses, conference papers, or book chapters) were not included in the review. We utilized both restricted vocabulary and free-text phrases to perform a thorough literature search. The search was conducted using Boolean operators “AND” and “OR” to combine the following terms: “polyphenols” OR “flavonoids” OR “flavanones” OR “flavonols” OR “isoflavonoids” OR “anthocyanidins” OR “chalcones” OR “stilbenes” OR “lignans” OR “phenolic acids” AND “depression” OR “depressive disorder” OR “depressive symptom” OR “antidepressant” OR “antidepressive”.

## 2. Characteristics of Polyphenols

Plants contain around 10,000 chemical substances that fall under the broad category of polyphenols. They are generated as byproducts of the metabolic process in response to free radicals or other environmental stressors [23]. Phenolic compounds have several roles in plants, such as protecting them from infections and oxidative damage, sensing light, affecting sensory properties, and controlling growth and reproduction [24]. Almost every food that is derived from plants contains polyphenols. Fruits, vegetables, seeds, grains, and nuts are the principal dietary sources of polyphenols for humans. Olives, chocolate, coffee, tea, red fermented vinegar, and wine are processed foods that also contribute to polyphenol intake [25,26]. The structural arrangements of polyphenols affect their absorption, metabolism, bioavailability, and biological activity. The four primary types of polyphenols are flavonoids, phenolic acids, lignans, and stilbenes [23,24]

**Figure 3:**
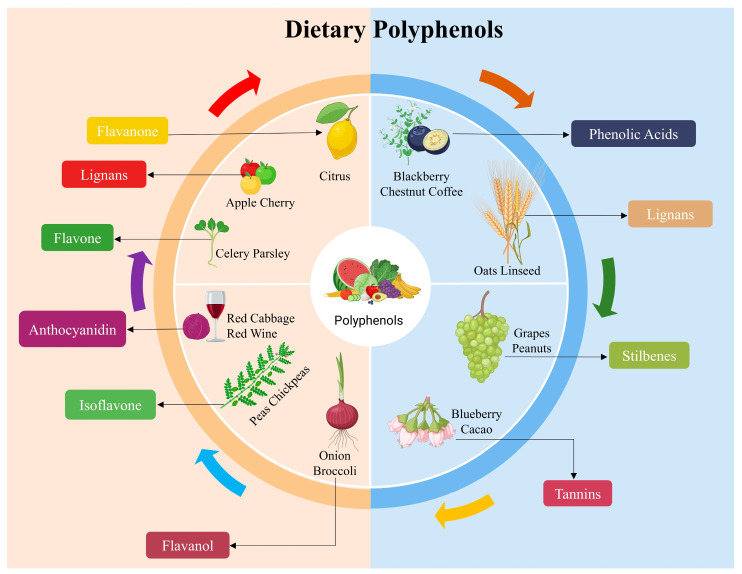
Sources of dietary polyphenols and their classifications.

More than four thousand kinds of plants contain flavonoids, the most common type of polyphenols. The vivid hues seen in plants, fruits, and vegetables are mostly due to them [27]. Kale, tomatoes, onions, apples, berries, herbal tea, and red wine all contain these chemicals in high amounts [28]. Soybeans also contain isoflavones, while anthocyanidins are found in berries, red cabbage, eggplant, and other colorful fruits and vegetables; catechins are found in dark chocolate, green tea, and red wine; flavones like apigenin, luteolin, and baicalin in cereals, aromatic herbs, and green and black tea; and naringenin and hesperidin, which are phenolic acids like gallic, caffeic, and ferulic acid, account for nearly 30% of the total polyphenols in our diet [23]. Foods such as red fruits, onions, and black radishes contain these acids [29]. Linseed, whole grains, and cereals are good sources of lignans, a small class of phenolic compounds [26]. The stilbene resveratrol is essential for human health and can be found mostly in red wine, peanuts, blueberries, cranberries, and grape skins [28]. Only recently in the last two to three decades, extensive research has shown that these phenolic chemicals have favorable benefits on human health. Studies have shown that they can fight against microbes and fungi, as well as modulate inflammation, antioxidants, and the immune system. They may also help with diabetes, cancer, blood coagulation, and neurological disorders [30,31,32]. Figure 3 shows the several food sources of primary dietary polyphenols.

## 3. Metabolism of Polyphenols

Polyphenols are organic compounds that include a minimum of two hydroxyl groups and two phenyl rings in their molecular structure. The structure of polyphenols, which are secondary metabolites produced in plant tissues, protects them from oxidative damage, UV radiation, and infections [33,34,35]. Several factors can impact the composition and amount of polyphenols, including the plant’s maturity at harvest, the weather, infectious processes, and processing and storage after harvest [36]. Subclasses of bioactive phytochemicals known as polyphenols include stilbenes, phenolic acids, flavonoids, and lignans [37,38].

Quercetin, hesperidin, isoflavones, anthocyanins, and flavan-3-ols all have a chroman ring attached to a second aromatic ring [39,40,41,42]. The amount of a plant compound known as a flavonoid that a human body can absorb is dependent on the moiety of the compound, which can be either a glycoside or non-glycoside [33,43]. Phase II enzymes convert dietary flavonoids from their hydrolyzed state to their glucuronide/sulfate form in the liver and epithelial cells [44,45]. Subsequently, the absorption of flavonoids occurs within the intestinal tract. The majority of flavonoids are delivered to the large intestine from the small intestine, where the colonic bacteria further break down deconjugated metabolites and aglycons. This makes it easier to absorb compounds like phenolic acids [33,44]. Intestinal bacterial flora metabolizes quercetin-3-O-rutinoside, an antioxidant derived from tomatoes, mainly in the large intestine, which also contains several glucuronidation and methylation products [43]. The microorganisms in the colon break down green tea flavan-3-ols even further into phenolic acids after they have been mainly digested in the small intestine [46]. The absorption of many flavonoids and their bioavailability in the systemic circulation is influenced by phenolic and polyphenolic chemicals, which are essential for gut bacteria. In addition, polyphenols spend more time in the colon than in the small intestine, giving them a great opportunity to affect the microbiota and health of the colon [47].

In humans, polyphenols may prevent or stop several age-related diseases. [48,49,50]. Researchers have shown in vitro that polyphenols’ health advantages stem from their direct antioxidative actions. However, it is possible that these antioxidative effects do not matter in humans because the concentrations of free radical scavengers achieved after oral consumption are not high enough to affect most tissues significantly [33,51,52,53]. However, their effects may be due to a plethora of other biochemical and molecular processes mediated by many intra- and intercellular signaling pathways, including the control of nuclear transcription factors, the regulation of fat metabolism, and the production of inflammatory mediators (e.g., cytokines, peptides, glycoproteins) [54,55]. Through downstream signals, flavonoids contribute to glucose homeostasis by, among other things, enhancing beta-cell proliferation and decreasing apoptosis, insulin resistance, inflammation, and oxidative stress in several cell types. Dihydrochalcone phlorizin from apple has the potential to alleviate hyperglycemia by selectively blocking the intestinal sodium-dependent glucose transporter 1 (SGLT-1) and the renal sodium-dependent glucose transporter 2 (SGLT-2) [56]. In particular, polyphenolic substances have protective effects against metabolic disorders and chronic illnesses, and multiple systematic investigations have shown that they also have anti-inflammatory and antioxidative properties [57]. Polyphenols may boost immunity, reduce cellular inflammation, and stop tumor angiogenesis in its tracks [58,59]. They have antioxidative properties that may account for most of their disease-prevention potential but that also cause epigenetic alterations and specific pharmacologic actions [60,61].

## 4. Behaviors Indicative of Depression

Many factors might set off depression, which is a mood disorder, including genes, childhood adversities, grief in the family, relationship difficulties, difficulties at work, and social issues [62,63]. Many lines of evidence point to depression as a systemic disease because of the far-reaching effects it has on oxidative stress [62,64]. Depression is the second leading cause of disability, behind myocardial infarction, according to the WHO. Women, in particular, are more likely than men to experience major depressive disorder, which affects an estimated 340 million individuals worldwide [65]. Depression encompasses an extensive neural network that integrates multiple areas, including cortical regions such as the prefrontal cortex and hippocampus, as well as subcortical areas like the hypothalamus [66], unlike neurodegenerative disorders, which are characterized by lesions in certain areas of the central nervous system Many different types of depression can be distinguished according to their symptoms, severity, and age of onset. These include major depressive disorder, premenstrual dysphoric disorder, persistent depressive disorder (dysthymia), and disruptive mood dysregulation disorder [67].

Many potential physical and mental factors might contribute to depression, but one of the most important is the accumulation of negative emotions, thoughts, and experiences throughout a person’s lifetime. In addition, some people are born with a higher susceptibility to depression than others [68]. Some genes appear to be associated with a higher risk of developing depression. It has been found that individuals whose levels of the neuronal transporter gene SLC6A15 are abnormal in some areas of the brain that are responsible for controlling emotions are at a higher risk of developing depression or other emotional problems associated with stress [69]. Typical signs of depression include low mood, anhedonia (lack of interest or enjoyment from pleasurable activities), lethargy, lack of energy, anxiety, trouble sleeping (hypersomnia or insomnia), changes in appetite and fluctuating weight, low self-esteem, and a lack of desire to have sexual relations [70]. Cognitive symptoms include mental enumeration, indecision, problem-solving difficulties, negative and catastrophic thinking, excessive self-criticism, and over-evaluation. One possible explanation for these symptoms is the correlation between depression and monoamine neurotransmitters, which include serotonin, noradrenaline, and dopamine. The neurotransmitters dopamine, noradrenaline, and serotonin work in tandem, and this leads to a synaptic change in depressed patients [71]. There may be a correlation between low noradrenaline levels and lethargy and a lack of focus, energy, and enthusiasm for life. A decrease in serotonin leads to compulsions, obsessions, and anxiety; a decrease in dopamine from the prefrontal cortex causes a decrease in motivation and pleasure [72]. People suffering from major depressive disorder were found to have lower dopamine levels and higher dopamine and serotonin transporter levels [73,74].

A relationship has been clearly established between depression and immunological mechanisms and the biological substances called cytokines [75]. Interleukin (IL)-1, IL-2, IL-6, and tumor necrosis factor-alpha (TNF-α) levels are significantly higher in depressed individuals [76,77]. Depression is associated with a shrinkage of the prefrontal cortex and hippocampus because neurogenesis is inhibited by an inflammatory immune response [78]. In addition to playing a significant role in mood regulation, cytokines mediate innate and adaptive immunity via the humor. Pro-inflammatory cytokines indeed elevate the HPA axis, leading to a significant rise in blood glucocorticoid levels [79]. Persistent hyperactivity of the HPA axis and dysfunction of the autonomic nervous system are hallmarks of severe depression in particular [80]. In particular, HPA activation triggers the release of cortisol and reactive oxygen species (ROS) by activating the adrenal medulla to create catecholamines and activating the hypothalamic production of corticotropic-releasing hormone [77]. This exemplifies the connection between oxidative stress and symptoms similar to those in depression [81]. When it comes to the physiopathology of major depressive disorder, glutamate is another key mediator. Memory, cognition, learning, and neural network training are all impacted by this excitatory neurotransmitter. An increase in the inflow of calcium and nitric oxide (NO) is caused by an overstimulation of N-methyl-D-aspartate (NMDA) receptors, which, in turn, is caused by elevated glutamate levels [82].

In addition, the anterior cingulate cortex was found to have a role in modulating emotional behavior in cases of depression [83]. The brain-derived neurotrophic factor (BDNF) is the main neurotrophin responsible for neurogenesis. Serum BDNF levels were found to be lower in depressed individuals. On the other hand, BDNF levels in the blood rose during antidepressant treatment. Decreased serum BDNF is associated with a number of mental diseases, but its associations with depression and the efficacy of antidepressants have piqued the public’s curiosity [84]. Depression and Alzheimer’s disease have comparable pathophysiological processes, such as impaired BDNF, compromised transforming-growth-factor-β1 (TGF-β1) signaling, and abnormal TNF-α signaling. Indeed, multiple pieces of evidence have drawn attention to the correlation between Alzheimer’s disease and depression [85]. An anti-inflammatory cytokine known as TGF-β1 plays an important role in memory formation and synaptic plasticity, and it also supports the protection against neurodegeneration caused by amyloid-β. It is worth noting that the severity of depression is associated with a decrease in TGF-β1 plasma levels in patients suffering from severe depression. Additionally, the regulatory cytokines Th1 and Th2 that TGF-β produces may have a positive impact on the subsequent response to antidepressant treatment [86,87].

Medications and non-medication approaches are frequently used in the treatment of depression. One type of antidepressant is an irreversible inhibitor of the monoamine oxidase (MAO) enzyme, which is responsible for the metabolism of monoamines [88]. By binding preferentially to the serotonin transporter (SERT or 5-HTT), selective serotonin reuptake inhibitors (SSRIs) increase serotonergic transmission and decrease muscarinic, adrenergic, and serotoninergic receptor levels [89]. Another category of antidepressants is tricyclic antidepressants (TCAs), which block the reuptake of serotonin and noradrenaline by binding to their respective receptors [90]. By binding selectively to the norepinephrine carrier and inhibiting norepinephrine reuptake, selective noradrenaline reuptake inhibitors (NARIs) prolong the amount of time that the neurotransmitter spends in the synaptic spineThe noradrenergic and specific serotonergic antidepressants (NaSSAs) have a dual mechanism of action that works as an antagonist on the alpha-2 adrenergic receptors and blocks the 5-hydroxytryptamine (5HT) serotonin receptors, thereby, increasing the levels of noradrenaline and serotonin in the synaptic cleft [91]. On the other hand, SNRIs block the reuptake of serotonin and norepinephrine, whereas SSRIs selectively block the reuptake of serotonin [92]. The last group of antidepressants is known as NDRIs, and they work by blocking the reuptake of noradrenaline and dopamine alone [93]. Similar to any other medications, the majority of antidepressants also present with unwanted side effects, such as gastrointestinal (GI) upset, headache, nausea, vomiting, change in appetite, and erectile dysfunction [94]. Researchers are interested in the natural substances found in fruits and vegetables because of their functional qualities and how quickly they start working with minimal side effects.

## 5. The Protective Role of Polyphenols Against Depression

Although the exact mechanisms by which naturally occurring flavonoids alleviate depression remain unclear, they have shown promising results. As a general rule, flavonoids are thought to function as analogs to antidepressants by modifying energy metabolic parameters, influencing behavior, reducing oxidative stress, and inhibiting overactive apoptosis (through modulating the expression of the Bax and Bak proteins) [19,95]. The capacity of flavonoids to interact with molecular and physiological mechanisms is a contributing factor to their influence on depression [19,95,96]. High-enough quantities of reactive metabolites and quantities of flavonoids are thought to activate kinases, transcription factors, and receptors in the brain [97]. Since the precise location of these flavonoids’ interactions with specific phytoconstituents’ intracellular signaling pathways is still unclear, the available information implies that they may exert a wide variety of effects. Flavonoids mainly owe their depressive and neuroprotective benefits to their antioxidative action [95,96,97]. In addition to their antioxidative properties, studies have shown that flavonoids may have multiple antidepressive effects; these include modulating the expression of proteins involved in neurotransmission and BDNF levels, increasing neuronal growth, inhibiting the activity of certain enzymes (such as MAO and acetylcholinesterase), modulating the channels for calcium and potassium ions, maintaining brain plasticity, and preventing the potential dissipation of the mitochondrial membrane [19,22,95].

### 5.1. Antioxidative Effects

A potential link between oxidative stress and the onset of depression is its ability to damage brain cell structure and function by upsetting the delicate equilibrium between oxidative and antioxidative defenses [11,98]. The antioxidative activity of some dietary natural products and minerals reduces behaviors similar to those in depression, according to multiple studies. In one study, lycopene enhanced neuroblastoma cell protection against oxidative stress and endoplasmic reticulum stress-induced damage by lowering 8-hydroxydeoxyguanosine (MDA), protein carbonyl, and 8-hydroxydeoxyguanosine (PKG) expression levels and by blocking the PERK signaling pathway [99]. Packed with phenols, anthocyanins, vitamin C, and flavonoids, the *Grewia asiatica* berry is a beloved fruit in Pakistan, which is enjoyed most often in carbonated beverages and fresh juices [100]. By elevating the levels of glutathione peroxidase (GPx) and superoxide dismutase (SOD), the berry juice of the *Grewia asiatica* plant may alleviate symptoms of depression [101]. The ethanol extract of *Saccharina japonica* was found in another investigation to increase SOD activity, which, in turn, reduced dextran sodium sulfate-induced depression in mice [102]. Furthermore, maqui berries improved the activities of superoxide dismutase (SOD) and catalase (CAT) and upregulated expression levels of reduced glutathione (GSH), which led to an antidepressive impact against post-stroke depression [103]. In addition, by improving Nrf2-mediated antioxidative defense, fish oil helped to prevent depression in elderly MRL/lpr mice [104]. To summarize, preclinical models have shown that lycopene, maqui berry, fish oil, *Grewia asiatica* berry juice, and *Saccharina japonica* reduce depression through antioxidative action; these findings warrant more investigation in humans.

### 5.2. Anti-Inflammatory Effects

Depressive disorder risk factors include neuroinflammation [105]. The activation of microglial cells has the potential to cause neuroinflammation and an upregulation of inflammatory cytokines, both of which can damage neurons and speed up the development of depression [106]. Results showed that n-3 polyunsaturated fatty acids (PUFAs) reduced levels of interleukin (IL)-6, IL-1β, TNF-α, and prostaglandin E-2, which helped alleviate postmenopausal depression caused by intermittent mild stress and mother separation [107]. Furthermore, an ethanol extract of the East Asian sea vegetable *Saccharina japonica* reduced the depressive symptoms manifested in mice subjected to dextran sodium sulfate by elevating levels of anti-inflammatory cytokines and decreasing the expression of nuclear factor kappa-B (NF-κB), NOD-like receptor 3, and Toll-like receptor-4 (TLR-4) transcripts [108]. In addition, the miR-22-3p/sirtuin 1 (SIRT1) signaling pathway was linked to a reduction in neuroinflammation and neuronal death, which, in turn, ameliorated depressive-like behaviors produced by lead acetate [103]. By reducing the expression levels of vascular cell adhesion molecule (VCAM), macrophage chemoattractant protein 1 (MCP-1), IL-6, IL-8, and TNF-α in the ileum and colon of mice, the oral administration of *Prevotella histicola* ameliorated estrogen-deficiency-induced depression in an ovariectomy mouse model [109]. Also, by reducing IL-1α, IL-6, and TNF-α expression and suppressing NF-κB activity in the hippocampus, *Lacticaseibacillus paracasei* NK112 protected against depression caused by *Escherichia coli* [110]. By lowering levels of IL-6 and nitric oxide (NO) and caspase-3 and caspase-9 mRNA expression in the hippocampus, epigallocatechin gallate (EGCG) had an antidepressant effect in a rat model of chronic unexpected mild stress (CUMS)-induced depression [111]. In the future, clinical research should confirm that *Saccharina japonica*, apple phenolic extract, *Prevotella histicola*, *Lacticaseibacillus paracasei* NK112, n-3 PUFA, and EGCG have an anti-inflammatory effect that reduces depression.

## 6. Controlling the Synthesis of Monoamine Neurotransmitters

A major part of treating depression is adjusting monoamine neurotransmitter systems, which is based on the monoamine hypothesis, one of the most widely held theories about the cause of depression [112]. Some minerals and natural products in the diet may control the generation of monoamine neurotransmitters, which may alleviate sadness, according to large-scale research. By increasing the levels of dopamine, serotonin, and norepinephrine, dehulled sunflower seeds alleviated the depressive-like behaviors observed in CUMS-induced animals [113]. By lowering colon serotonin levels while raising the hippocampal levels, EGCG ameliorated depression-like behaviors brought on by CUMS, according to another study [114]. Additionally, research has shown that navel orange essential oil can increase levels of the feel-good neurotransmitters dopamine and serotonin, which, in turn, can alleviate depression [115]. Additionally, by increasing the expression levels of dopamine, serotonin, and norepinephrine in the hippocampus, fermented milk made from adzuki bean sprouts may alleviate moderate depressive symptoms [116]. Furthermore, n-3 PUFA increased serotonin levels in the brainstem and serotonin-1A receptor expression in the hippocampus, which, in turn, prevented depression [107]. Dehulled sunflower seeds, essential oil of navel orange, fermented milk from adzuki bean sprouts, EGCG, and n-3 PUFA all showed antidepressive effects by modulating the production of monoamine neurotransmitters; however, human clinical trials are needed to confirm these effects and mechanisms.

### 6.1. Enhancing Neurotrophin Synthesis

A family of structurally and functionally related proteins known as neurotrophins—including BDNF, nerve growth factor, neurotrophin-3, and neurotrophin-4—are essential for neuronal survival, development, and function [117]. Research suggests that certain natural foods may alleviate depression by boosting the body’s natural synthesis of neurotrophins. By increasing the expression levels of cyclic adenosine monophosphate response-element-binding protein (CREB), protein kinase B, and BDNF in the hippocampus, garlic essential oil demonstrated an antidepressive effect against CUMS-induced depression [118]. In addition, the Asian medicinal plant *Geum japonicum* is well liked. By increasing BDNF expression in the hippocampus, it demonstrated neuroprotective effects on mice who were depressed due to corticosterone (CORT). It also reduced the neurotoxicity that SH-SY5Y cells experienced as a result of CORT [119]. Conjugated linoleic acid and fish oil both enhanced brain BDNF and synaptic protein expression in a depression paradigm involving lupus-prone MRL/lpr elderly mice [104]. Furthermore, by inhibiting monoamine oxidase activity and increasing expression levels of monoamine neurotransmitters, BDNF, and tyrosine kinase receptor B (TrkB) [120], pure anthocyanin from purple cauliflower ameliorated depressed symptoms in CUMS-induced rats. By elevating expression levels of neurotrophins, including BDNF and neurotrophin-3, sesamin alleviated depressive symptoms in a mouse model of CUMS-induced depression [121]. In addition, one study discovered that rats with epilepsy exhibited less depressive-like behaviors after taking probiotic supplements, which were attributed to an increase in nerve growth factor and BDNF expression levels [122]. Resveratrol improved depressive symptoms in rats with male hypogonadism by elevating BDNF and neurotrophin-3 levels in the hippocampus and frontal cortex, respectively [123]. Clinical trials should be conducted to further examine the effects and mechanisms of *Geum japonicum*, garlic essential oil, fish oil, conjugated linoleic acid, anthocyanin, and nervous growth factors on depression.

### 6.2. Reducing Excessive HPA Axis Activity

An integral aspect of the neuroendocrine system, the HPA axis regulates the stress response and mediates mood [124]. Symptoms of depression were exacerbated by an increase in corticotropin-releasing factor (CRF) and adrenocorticotropic hormone (ACTH) levels brought on by an overactive HPA axis, which blocked the negative feedback signal of cortisol [125]. To alleviate depressive symptoms brought on by chronic stress and lipopolysaccharide (LPS), it was shown that phospholipids rich in eicosapentaenoic acid (EPA) reduced hyperactivity of the HPA axis [126]. Furthermore, by enhancing neurotransmitter synthesis and transport and decreasing hyperactivation of the HPA axis, saponin compounds isolated from Baihe Zhimu Tang, a traditional Chinese medication, had an antidepressive effect [127]. By lowering blood levels of ACTH and CRF, decreasing brain expression of miRNA-218 and CRF, and increasing expression of the glucocorticoid receptor, n-3 PUFA exerted antidepressive-like effects in a rat model of postmenopausal depression induced by chronic mild stress and maternal separation [107]. In addition, research has shown that n-3 PUFA helps to regulate the HPA axis, which, in turn, reduces circulating ACTH and CORT levels and downregulates the production of hypothalamic CRF, which alleviates pup separation-induced postpartum depression [128]. Another common dietary supplement, royal jelly, reduced CUMS-induced depression by blocking adrenal gland CORT production [129]. To summarize, clinical trials should confirm that royal jelly, EPA-enriched phospholipids, saponin compounds, and n-3 PUFA alleviate depressive symptoms by reducing hyperactivity of the HPA axis as seen in animal models.

### 6.3. Axis Modulation Between the Microbiota, Gut, and Brain

It is now widely acknowledged that gut microbiota plays a significant role in illness prevention and management [130,131,132,133]. Multiple studies have linked disturbances in the gut microbiota to inflammatory factor production changes, which regulate many signaling pathways linked to depression [134]. The HPA axis and monoamine neurotransmitter effectiveness are both impacted by gut microbiota abnormalities, which also heighten the permeability of the gut barrier, trigger immunological responses, and cause systemic inflammation [135]. Some probiotics may alleviate depressive symptoms, according to several studies. By increasing the number of gut flora, particularly of *Akkermansia* and *Lactobacillus*, the newly-emerging probiotic *Prevotella histicola* protected against estrogen-deficiency-induced depression [108,133]. In addition, via modifying the gut microbiota, elevating BDNF expression, and inhibiting BDNF–MAPK pathway activity, *Lactobacillus casei* had antidepressive effects on postpartum depression [136]. Furthermore, research has shown that *Lactobacillus casei* can alleviate CUMS-induced depression by modulating BDNF/TrkB signaling pathways and by correcting the gut microbiota’s structural alterations [137]. Another study found that *Bifidobacterium* E41 and M2CF22M7 reduced depressive symptoms by balancing gut microbes and increasing serotonin and BDNF gene expression levels [138]. Also, by controlling the makeup of the gut microbiota and alleviating constipation by raising the quantity of fecal fluids, *Lactobacillus kefiranofaciens* ZW3, which was isolated from Tibetan Kefir grains, may alleviate depressive symptoms [139]. Furthermore, regulating gut microbiota through vagus-nerve-mediated gut–brain communication and improving *Escherichia coli* K1-induced depression was achieved by *Lactobacillus gasseri* NK109 [140].

Not only do probiotics alleviate symptoms of depression, but other nutrients and natural products included in a healthy diet have the same effect by regulating the gut–brain axis. Increasing norepinephrine and serotonin expression levels, inhibiting neuroinflammation, promoting the formation of short-chain fatty acids, and reconstructing the gut microbiome were some of the mechanisms by which a high-fiber diet ameliorated postpartum depressive-like behaviors caused by antenatal obesity [141]. By elevating the relative abundance of *Burkholderiales* and *Bifidobacterium* and decreasing that of *Desulfovibrionales* and *Desulfovibrio*, chlorogenic acid had antidepressive effects in a rat model of ACTH-induced depression [142]. In addition, the study found that red ginseng fermented with *Bifidobacteria* protected against depression caused by *Escherichia coli*. This was achieved by increasing the number of Bacteroidetes, lowering the number of Proteobacteria, and upregulating the expression of BDNF through NF-κB [143]. Tea polyphenols were discovered to regulate the circadian rhythm and increase the quantity of probiotics to attenuate depressive symptoms [133]. Additionally, problems with the gut microbiota were connected with circadian rhythm disorders. Vitamin B3, in its most common form, nicotinamide riboside, is abundant in dairy products and yeast [144]. Nicotinamide riboside altered the gut microbiota composition in an alcohol-induced depression rat model, leading to elevated BDNF levels in the hippocampus and decreased production of inflammation-related cytokines [145]. Also, soy isoflavones alleviated CUMS rats’ depression symptoms, and researchers linked this to an increase in monoamine neurotransmitter levels and a diversification of the gut flora [146]. The phenolic acid compound coniferyl ferulate is primarily found in umbelliferous plants. It has been shown to protect against CUMS-induced depression by enhancing the gut microbiome’s reconstruction and reducing inflammation in the colon by downregulating the expression levels of IL-6, IL-1β, and TNF-α [147]. By influencing the microbiota–gut–brain axis, the traditional fermented food *Semen sojae praeparatum* showed antidepressive properties. To be more precise, *Semen sojae praeparatum* controlled the levels of serotonin, norepinephrine, GABA, and BDNF in the hippocampus and increased the abundance of the *Ruminococcaceae* species UCG-008 [148].

**Figure 4:**
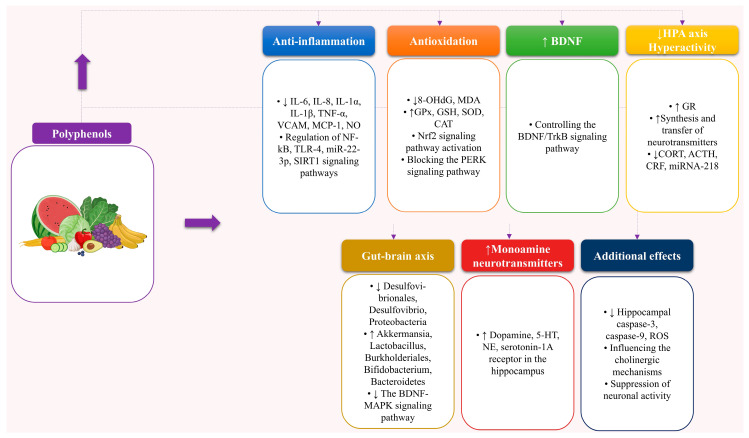
The impact and processes of natural food items on depression. ↑ denotes an increase, whereas ↓ signifies a decrease. Adrenocorticotropic hormone (ACTH); brain-derived neurotrophic factor (BDNF); CAT denotes catalase; CORT represents corticosterone; CRF indicates corticotropin-releasing factor; GPx denotes the enzyme glutathione peroxidase; GR denotes the glucocorticoid receptor, while GSH refers to glutathione. 5-HT denotes serotonin; IL refers to interleukin; MAPK denotes mitogen-activated protein kinase. MDA represents malondialdehyde; NE denotes norepinephrine; NF-κB refers to nuclear factor kappa-B; NO signifies nitric oxide; and MCP-1 indicates macrophage chemoattractant protein 1. Nrf2 denotes the nuclear erythroid-related factor 2. 8-OHdG represents 8-hydroxydeoxyguanosine; PERK refers to protein kinase-like endoplasmic reticulum kinase; ROS indicates reactive oxygen species; SIRT1 signifies Sirtuin 1; TNF-α denotes tumor necrosis factor-α; SOD indicates superoxide dismutase; TrkB represents tyrosine kinase receptor B; VCAM stands for vascular cell adhesion molecule; TLR-4 stands for toll-like receptor-4.

In a nutshell, preclinical models have shown that certain dietary natural products and nutrients, including *Lactobacillus casei*, *Bifidobacterium* E41 and M2CF22M7, *Lactobacillus kefiranofaciens* ZW3, *Lactobacillus gasseri* NK109, *Prevotella histicola*, *Semen sojae praeparatum*, *Bifidobacteria*-fermented red ginseng, dietary fiber, tea polyphenols, chlorogenic acid, nicotinamide riboside, soy isoflavones, and coniferyl ferulate could potentially alleviate depression by modulating the microbiota-gut-brain axis. More research in clinical trials is needed to determine the processes and potential effects of these drugs on depression. Consequently, it is reasonable to assert that numerous studies have demonstrated that certain dietary polyphenols exert protective effects against depression via various mechanisms, including via anti-inflammatory and antioxidative effects, enhancement of monoamine neurotransmitter production, and normalization of HPA-axis hyperactivity, regulation of the microbiota–gut–brain axis, among others, as summarized in Figure 4.

## 7. Research on the Antidepressive Effects of Polyphenols in Animal Models and Human Trials

Several studies have shown that certain natural products and dietary polyphenols in the diet can protect against depression. These effects can be attributed to various mechanisms, some of which are discussed in detail below (Table 1 and Table 2), such as anti-inflammatory effects, antioxidative effects, the promotion of monoamine neurotransmitter production, the normalization of the hyperactivity of the HPA axis, and regulation of the microbiota–gut–brain axis.

It has been found that flavonoids in the diet are associated with a lower risk of depression. Polyphenols were shown to have a substantial positive impact on depressive symptoms in the majority of the trials outlined above in Table 1 and Table 2. In some investigations, no significant benefit was observed following the intervention with polyphenols. On the other hand, certain studies examining the impact of polyphenols during menopausal transition found statistically significant improvements in perimenopause, menopause, or postmenopausal depressive symptoms. Also, in postmenopausal women with osteopenia, one clinical trial found a statistically significant effect on depressive symptoms. A few studies that looked at the effects of polyphenols on anxious people indicated that they significantly reduced depressive symptoms.

## 8. Constraints of Employing Polyphenols in the Treatment of Depression

The present analysis presents new evidence that polyphenols have antidepressive benefits. The research does, however, need additional inquiry into several significant evidentiary gaps. In their evaluations of the effects of polyphenols and meals high in polyphenols, several studies in animals and humans failed to account for the total amount or the kind of polyphenols consumed. Polyphenol consumption was not evaluated in any of the animal trials at the dietary pattern or whole-diet levels. These studies [191,192,193] fail to consider the complexity of dietary polyphenol exposure, which includes interactions between polyphenols and nutrients, the effects of multiple polyphenols added together, and the variability in polyphenol bioavailability among individuals. The probable link between polyphenols and depression risk and the polyphenol effects on depressed symptoms may be better understood if research evaluated polyphenol consumption at the level of dietary patterns rather than at the level of individual foods.

The composition of an individual’s gut microbiome has a significant impact on polyphenol bioavailability [194] because the gut microbiota plays a key role in the biotransformation of polyphenols into their bioactive metabolites [195]. The disparities in the outcomes of the research that were part of this meta-analysis could be attributable, in part, to the fact that individuals’ gut microbiota compositions vary. Not many intervention studies have looked at important biomarkers for pathways that are involved, such as inflammation (e.g., C-reactive protein), oxidative stress (e.g., glutathione), and the kynurenine pathway (e.g., kynurenine and tryptophan) [194]. Potential mediating mechanisms in the link between polyphenol intake and depression should be taken into account in future intervention studies by considering the composition of the gut flora and associated biomarkers.

More comprehensive studies examining the action mechanism, activity level, and structure–activity relationship are required. The reason behind this is the diverse range of biological effects that polyphenols can have. This further suggests that polyphenol combinations may have a synergistic effect [196]. A better understanding of polyphenols, including how to administer them, which tissues they target, how much to take, and what kind of phenolic extracts work best, requires more research. None of the studies examined the potential relationships between flavonoid consumption, gender, and depressive symptoms. Neurochemical variables have a significant role in the pathophysiology of depression, but no clinical study has looked at the link between flavonoid intake and these factors. In addition, the role of social and cultural variables in the onset of depression symptoms was disregarded. Extensive dose testing is required in preclinical research to determine the highest safe single dose and the profile of long-term safety. Most people think polyphenols are harmless and non-toxic because they come from natural sources. Preclinical and clinical data show that the phenolic compounds under evaluation are safe, have no side effects, and have good tolerance [197,198,199,200,201,202]. Having said that, this cannot be made into a rule. There have to be additional studies on the total toxicity and amount of hazardous chemicals made during food processing or polyphenol extraction.

## 9. Perspectives and Concluding Remarks

Throughout the world, people’s quality of life has taken a hit due to the prevalence of psychiatric disorders like depression. Epidemiological studies have shown that some nutrients and food items, including fish, walnuts, coffee, tea, fruits, and vegetables, can help protect against depression. Numerous nutrient and natural-product-rich diets have been shown to have antidepressive effects in animal studies. These effects have been attributed to various pathways, such as modulation of the gut–brain axis and the HPA axis, regulation of monoamine neurotransmitter levels, amelioration of oxidative stress, and inhibition of inflammation. Studies on humans have shown that dietary nutrients and natural compounds such as EGCG, isoflavones, quercetin, rutin, chrysin, luteolin, resveratrol, apigenin, and genistein may have antidepressive effects. Further investigation is warranted into the causes and consequences of these disorders and the role of other dietary natural products and nutrients. There needs to be a greater number of clinical trials investigating the impact of dietary natural products and nutrients on human depression, as most of the current evidence comes from preclinical models. In addition, one should pay attention to the negative consequences. Another potential strategy for preventing and managing these diseases is the development of functional meals containing certain dietary natural products and nutrients that have antidepressive properties.

## Figures and Tables

**Table 1:** Summary of preclinical investigations assessing the functional characteristics of polyphenols in depressive behavior. The table enumerates the animal models employed in the investigations, the polyphenol compound utilized, the treatment types, the dosages administered, and the findings obtained.

Polyphenol	Study Type	Model	Dosage and Duration of Treatment	Effects and Mechanisms	Reference
EGCG	In vivo	CUMS rats	50 mg/kg	Exhibited antidepressive effects, decreased IL-6 and NO; reduced levels of caspase-3 and caspase-9	[111,149]
EGCG	In vivo	CUMS rats	50 mg/kg	Controlled symptoms of depression; decreased serotonin levels in the colon; enhanced serotonin levels in the hippocampus	[114,150]
Resveratrol	In vivo	Wistar–Kyoto male rats	40 mg/kg	Attenuated depressive symptoms; elevated brain-derived neurotrophic factor (BDNF) and neurotrophic factor-3 (NT3)	[123,151]
Soy isoflavones	In vivo	CUMS rats	40, 80, 160 mg/kg	Alleviated depression symptoms; raised the variety of bacteria in the digestive tract; elevated concentrations of monoamine neurotransmitters	[146,152]
Curcumin	In vivo	Wistar male rats	40 mg/kg	Decreased depressive symptoms; restrained severe synaptic degeneration; enhanced synaptic performance	[153]
Chlorogenic acid	In vivo	Wistar rats	500 mg/kg	Showed antidepressive effects; increased *Burkholderiales* and *Bifidobacterium* levels; reduced *Desulfovibrionales* and *Desulfovibrio* levels	[142,154]
Apple phenolic	In vivo	Kunming mice	200 ppm in normal saline	Improved depressive-like actions caused by lead acetate; lessened neuroinflammation and cell death in neurons; controlled the miR-22-3p/SIRT1 signaling pathway	[109,155]
Caffeine and caffeic acid	In vivo	Swiss albino mice	Caffeine: 15 mg/kg Caffeine + caffeic acid: 10 mg/kg + 5 mg/kg	Reduced symptoms of anxiousness; Reduced levels of inflammatory markers	[156]
Apigenin	In vivo	Male Sprague–Dawley rats	20 mg/kg for 21 days	Reduced IL-1 production; lowered levels of the NLRP3 inflammasome; increased expression of PPAR	[157]
Baicalein	In vivo	Male Sprague–Dawley rats	10, 20, and 40 mg/kg for 14 days	Reversed the decline in BDNF and dopamine levels in the hippocampus	[158]
Chrysin	Oral	Male C57B/6J mice	5 and 20 mg/kg for 14 days	Enhanced production of neurotrophic factor in the brain; elevated concentrations of serotonin in the hippocampus	[159]
Luteolin	Oral	Male ICR mice	50 mg/kg for 23 days	Reduced stress in the hippocampus by preventing the production of proteins associated with stress in the endoplasmic reticulum	[160]
Fisetin	Oral	Male ICR mice	20, 40, or 80 mg/kg for 7 days	Nutrient levels and iNOS messenger RNA levels were counteracted by regulating NF-κB; reduced the overexpression of proinflammatory cytokines (particularly IL-6, IL-1β and TNF-α) caused by LPS	[161]
Kaempferol	Oral	Male ICR mice	30 mg/kg/day for 14 days	Higher amounts of β-endorphins in the plasma or POMC mRNA in the hypothalamus	[162]
Myricetin	Intraperitoneal	Male C57BL/6 mice	50 mg/kg for 21 days	Raised levels of BDNF in the hypothalamus; decreased amount of free radicals	[163]
Quercetin	Oral	Male olfactory bulbectomy rats	40 and 80 mg/kg for 14 days	Protective effects on neurons through the microglial inhibitory pathway; neuroinflammation and apoptosis by oxidative and nitrosative stress were slowed down	[164]
Rutin	Oral	Male Sprague–Dawley rats	5 and 10 mg/kg for 14 days	Defense against oxidative stress	[165]
Hesperidin	Oral	Male C57BL/6 mice	50 mg/kg for 13 days	Enhanced levels of nerve growth factor and BDNF in the hippocampus, controlling cytokines that promote inflammation; persistent neuroplasticity; stimulation of acetylcholinesterase	[166]
Naringenin	Oral	Male ICR mice	5, 10 and 20 mg/kg for 14 days	Elevated levels of the neurotransmitters serotonin, dopamine, norepinephrine, and glucocorticoid in the hippocampus of the brain	[167]
Quercetin	Intraperitoneal	Albino Wistar mice	20 mg/kg for 14 days	Reduced stress-induced lipid peroxidation and elevated SOD, CAT, and GPx levels, causing antidepressive and anxiolytic effects; boosted Ach and decreased 5-HIAA levels in stressed mouse brains, improving serotonergic and cholinergic functioning	[168]
Quercetin and resveratrol	Intraperitoneal	Wistar albino rats	30 mg/kg for 35 days	Reduced immobility and boosted sucrose consumption, reducing depression. Both polyphenols increased SOD, CAT, and GSH levels in antioxidative defense and decreased MDA levels. They also decreased NF-κB and pro-inflammatory cytokine levels (IL-6, TNF-α, and IL-1β). Both polyphenols restored the HPA axis and lowered serum corticosterone levels	[169]

SOD, superoxide dismutase; CAT, catalase; GPx, glutathione peroxidase; Ach, acetylcholine; 5-HIAA, 5-hydroxyindoleacetic acid; IL, interleukin; TNF-α, tumor necrosis factor-α; NO, nitric oxide; NF-κB, nuclear factor-κB; GSH, glutathione; MDA, malondialdehyde; LPS, lipopolysaccharide; BDNF, brain-derived neurotrophic factor; HPA, hypothalamic–pituitary–adrenal.

**Table 2:** Summary of data from observational and clinical studies evaluating the impact of polyphenols on depressed symptoms. The table enumerates the subjects and sample size employed in the investigations, the polyphenol compound utilized, the treatment types, the dosages administered, and the findings obtained.

Polyphenol	Subjects	Sample Size	Dosage and Duration of Treatment	Result/Observation	Reference
Soy isoflavone	Adults aged 65 years and up	3999	Consumption varied from 4 to ≥19 mg per day	No correlation between isoflavones and depression	[170]
Flavonoid	Participants with a modest mental health issue (45–59 years old)	349	Consumption of 265 mg per day	No correlation between flavonoid consumption and depression	[171]
Isoflavone	Expectant mothers (mean age 31.2 years)	1744	Healthy pattern, 43.5 mg per day; Japanese pattern, 32.7 mg per day; Western pattern, 31.2 mg per day	Depressive symptoms during pregnancy were negatively correlated with healthy and Japanese patterns	[172]
Soy isoflavone	Expectant mothers (mean age 31 years)	1745	Consumption varied from 10.5 to 49.4 mg per day	Isoflavone intake was linked to decreased depression rates during pregnancy across all groups	[173]
Flavonoid	Participants (aged 18–92) who took part in the Mediterranean Healthy Eating, Lifestyle, and Aging Study	1572	Consumption varied from 157.0 to 543.7 mg per day	Higher flavanone and anthocyanin intake was linked to lower rates of depression	[174]
Soy isoflavone	Men aged between 19–83 years	1335	Consumption varied from ≤10.61 to ≥25.79 mg per day	A high intake of isoflavones was linked to decreased rates of depression in all groups	[175]
Flavonoid	Women participants in the Nurses’ Health Study (with a mean age of 67 years at baseline) and NHSII (with a mean age of 47 years at baseline)	82,648	Consumption varied from 127.6 to 779.4 mg per day	The high-flavonoid-consumption group showed a reduction in depression risk compared with the lowest intake group. The strongest connections were found with flavones and proanthocyanidins	[176]
Green tea	Participants with a mean age 25.7 years	46	Green tea (400 mg per day; EGCG 45.6%; epigallocatechin 16.7%; epicatechin-3-gallate 11.4%; epicatechin 6.8%) [35 days]	Significant effect (treatment vs. control)	[177]
Asperugo procumbens capsules	Participants aged between 18–70 years with mild depression	25	1.2 g/d; 6 mg total flavonoids [42 days]	Significant lower effect vs. antidepressant	[178]
Ginko biloba tablets	Participants with a mean age of 82.8 years and a history of age-related memory loss	123	(160 or 240 mg/d; 24% ginkgo flavonols) [168 days]	Insignificant effect	[179]
Isoflavone tablets	Women (46–55 years old) experiencing vasomotor symptoms of menopause	100	(60 mg/d) [90 days]	Significant effect (treatment vs. control)	[180]
EGCG capsules	Participants aged 18–50 years who are overweight	116	(50 mg/d) [60 days]	Significant effect (treatment vs. placebo)	[181]
EGCG capsules	Participants (48.2 years old on average) diagnosed with multiple sclerosis	46	(800 mg/d) with coconut oil (60 mL/d) [120 days]	Significant effect vs. baseline	[182]
Flavonoid-rich orange juice	Comparing a depressed and non-depressed population (participants’ mean age 21.8 years)	40	(380 mL/d; 600 mg flavonoids) [56 days]	Significant effect vs. baseline	[183]
EGCG capsules	Individuals diagnosed with bipolar disorder and schizophrenia who are at least 18 years old	25	(N/A mg/d; capsules enriched with 150 mg of theaflavin) [56 days]	Insignificant effect	[184]
Isoflavone capsules	Women having postmenopausal symptoms (mean age 53.5 years)	109	(80 mg/d; extract in form of biochanin A, formononetin, genistein and daidzein) [90 days]	Significant effect in the Hospital Anxiety and Depression Scale and Zung Self-Rating Depression Scale	[185]
Soy isoflavone capsules	Women with menopause	20	(35 or 70 mg/d isoflavones) [42 days]	Significant effect in 35 mg groups vs. baseline	[186]
Cocoa extract	Participants with obesity (mean age 57 years)	37	(1.4 g/d; 414 mg flavan-3-ols; 153 mg epicatechin; 15 mg catechin; 246 mg procyanidins) [28 days]	Significant effect vs. baseline	[187]
Genistein tablets	Women having postmenopausal symptoms and osteopenia (49–67 years old)	262	(54 mg/d) [730 days]	Significant effect (treatment vs. placebo)	[188]
Chamomile capsules	Participants(mean age 45.7 years) suffering from anxiety and/or depression	179	(1500 mg/d; 18 mg of flavonoids) [56 days]	Significant effect in individuals with depression	[189]
Chamomile capsules	Individuals with mild depression (mean age 42.9 years)	41	(220 mg/d; 1.2% apigenin) [56 days]	Significant effect (treatment vs. placebo)	[190]
